# Dispositional Affect in Unique Subgroups of Patients with Rheumatoid Arthritis

**DOI:** 10.1155/2016/1024985

**Published:** 2016-03-03

**Authors:** Danielle B. Rice, Swati Mehta, Janet E. Pope, Manfred Harth, Allan Shapiro, Robert W. Teasell

**Affiliations:** ^1^Lawson Health Research Institute, London, ON, Canada N6C 0A7; ^2^St. Joseph's Health Care, Parkwood Institute, London, ON, Canada N6C 0A7; ^3^Western University, London, ON, Canada N6A 3K7; ^4^Schulich School of Medicine and Dentistry, Western University, London, ON, Canada N6A 3K7

## Abstract

*Background*. Patients with rheumatoid arthritis may experience increased negative outcomes if they exhibit specific patterns of dispositional affect.* Objective*. To identify subgroups of patients with rheumatoid arthritis based on dispositional affect. The secondary objective was to compare mood, pain catastrophizing, fear of pain, disability, and quality of life between subgroups.* Methods*. Outpatients from a rheumatology clinic were categorized into subgroups by a cluster analysis based on dispositional affect. Differences in outcomes were compared between clusters through multivariate analysis of covariance.* Results*. 227 patients were divided into two subgroups. Cluster 1 (*n* = 85) included patients reporting significantly higher scores on all dispositional variables (experiential avoidance, anxiety sensitivity, worry, fear of pain, and perfectionism; all *p* < 0.001) compared to patients in Cluster 2 (*n* = 142). Patients in Cluster 1 also reported significantly greater mood impairment, pain anxiety sensitivity, and pain catastrophizing (all *p* < 0.001). Clusters did not differ on quality of life or disability.* Conclusions*. The present study identifies a subgroup of rheumatoid arthritis patients who score significantly higher on dispositional affect and report increased mood impairment, pain anxiety sensitivity, and pain catastrophizing. Considering dispositional affect within subgroups of patients with RA may help health professionals tailor interventions for the specific stressors that these patients experience.

## 1. Introduction

Rheumatoid arthritis (RA) is an autoimmune disease characterized by joint swelling and tenderness at multiple sites in the body. These symptoms have a disabling effect on an individuals' mental and physical health [1]. An international study examining data from 32 countries, the QUEST-RA study, found that more than a third of patients reported work related disability due to RA [2]. Furthermore, health care costs of RA management remain high even after major advancements in treatment. Hallert et al. (2014) estimated a mean total cost of EUR 14,768 per patient in their first year of being diagnosed with RA and EUR 18,438 per year by year six [3].

Individuals with RA experience significant levels of chronic pain that negatively impacts multiple quality of life domains [4]. The related disability has been linked to several psychological contributors including depression, anxiety, and stress [5]. Epidemiological and clinical studies have consistently revealed a higher prevalence of depressive and anxiety disorders in patients with RA than in the general population [1, 6–8]. Presence of psychiatric symptoms among RA individuals has been shown to increase the perception of pain, use of analgesics, and work disability [7]. The comorbidity between chronic pain and depression has been established among several studies and management strategies have been implemented in clinical practice guidelines [9].

However, aspects of personality are also increasingly being viewed as important by pain researchers, clinicians, and patients with chronic pain. Newth and Delongis (2004) found that personality was a strong moderator of coping after chronic pain in RA individuals [10]. Specific personality traits such as neuroticism predicted both day to day reports of illness symptoms and the subsequent accuracy with which symptoms are recalled over the same period [11]. A recent review concluded that specific dispositional variables including neuroticism, anxiety sensitivity, and experiential avoidance can predispose individuals with chronic pain to use ineffective strategies in coping [12]. Other studies looking at general chronic pain populations have found dispositional variables including maladaptive perfectionism [13], experiential avoidance [12, 14], anxiety sensitivity [15, 16], and psychological inflexibility [17] negatively related to patients clinical outcomes including mood and disability.

These dispositional variables have also been discussed in a qualitative study that included eight overactive chronic pain patients [18]. All patients believed that their tendency to do too much was related to their personality and five of the eight participants noted that their over activity resulted in depressed mood, anxiety, and/or irritability. These patients identified aspects of psychological inflexibility including experiential avoidance and reported being perfectionists and unable to relax and described themselves as having obsessive personality traits [18]. To the best of the authors' knowledge these aspects of personality have not yet been studied specifically in patients diagnosed with RA, even though these patients experience unique difficulties in comparison to patients with a diagnosis of chronic soft tissue pain [19]. Examination of specific dispositional variables and how they affect clinical outcomes among individuals with RA may be important in screening patients at risk for developing more optimized management plans.

The present study represents a preliminary step in identifying subgroups among persons with RA based on dispositional affect. The aims of the study were to use a cluster analysis to identify homogenous pain behavior subgroups among persons with RA through a number of dispositional personality variables that have been previously linked to maladaptive coping styles [12]. The secondary aim was to determine if the subgroups identified differed on measures of mood, pain catastrophizing, fear of pain, disability, and quality of life.

## 2. Methods

### 2.1. Participants

Participants included patients with RA diagnosed by a rheumatologist (using the American College of Rheumatology Criteria) that scheduled a regular outpatient clinic appointment and were recruited over a 20-month period from an academic rheumatology clinic in London, Ontario (St. Joseph's Health Care London, associated with Western University). Patients at least 18 years who had a diagnosis of RA and self-reported pain secondary to RA for greater than three months were eligible for inclusion. Given that this study involved the completion of questionnaire booklets, exclusion criteria included the inability to read and write in English. Ethics was reviewed and approved by the Office of Research Ethics at the University of Western Ontario in London, Ontario, Canada. All eligible participants signed informed consent prior to completing any questionnaires for the study.

### 2.2. Procedures

Patients who met the inclusion criteria and agreed to participate were referred to the research coordinator by their primary physician. The research coordinator provided potential participants with the letter of information and consent form. Patients were made aware that their decision to participate in the study would in no way interfere with their standard care at the hospital. All patients received individualized pharmacotherapy and psychotherapy or referrals as seen fit by the multidisciplinary team. Eligible participants were mailed a package introducing the study two weeks prior to their scheduled clinic appointment with their rheumatologist. The package contained the study information letter, a consent form, and the first of two questionnaire booklets. Research assistants followed up with phone calls to all eligible patients to explain the procedures of the study, answer any study related questions, and confirm that the patient was still experiencing pain secondary to RA. Consenting participants completed the first booklet of questionnaires regarding demographics (age, gender, years of education, and relationship status), time since RA diagnosis, and average pain intensity prior to their clinic appointment. Participants were asked to arrive half an hour early to their clinic appointment to provide research assistants with their first questionnaire booklet and complete the second booklet questionnaires that included measures of dispositional affect, pain catastrophizing, fear of pain, quality of life, and disability. One researcher independently entered questionnaire responses into a SPSS database which was then validated by a second researcher.

### 2.3. Demographic Measures

Demographic variables including age, sex, years of education, marital status, and years since RA diagnosis were assessed with single straightforward patient-report items.

#### 2.3.1. Average Pain Intensity Rating

Pain ratings for current, least, average, and worst pain were summed to yield an aggregate pain intensity score.

### 2.4. Cluster Variable Measures

#### 2.4.1. Acceptance and Action Questionnaire (AAQ)

The AAQ [20] is a 9-item measure of experiential avoidance, that is, an unwillingness to remain in contact with distressing private experiences (body sensations, emotions, and thoughts) and the inclination to alter the form or frequency of these experiences. It yields a single factor solution and is correlated with a wide range of negative behavioural and physical health outcomes [20].

#### 2.4.2. Anxiety Sensitivity Index (ASI)

The ASI [21] is a 16-item measure of the fear of anxiety-related symptoms comprised of three factors: fear of the somatic symptoms of anxiety; fear of mental incapacitation (cognitive dyscontrol); and fear of negative social repercussions of anxiety [20]. These factors can be summed for a total score. Each item is rated on a five-point Likert scale ranging from 0 (very little) to 4 (very much). The instrument's psychometric properties and predictive validity have been well established [22, 23].

#### 2.4.3. Frost Multidimensional Perfectionism Scale (FMPS)

The FMPS [24] contains subscales measuring six different dimensions of perfectionism. In the present study, we used the total score with the parental standards and criticism subscales omitted. Research suggests that the concerns about mistakes and doubts about actions subscales are related to negative affectivity and reflect “maladaptive” perfectionism, while the high standards and need for organization subscales are unrelated or negatively related to negative affectivity [25–27].

#### 2.4.4. Penn State Worry Questionnaire (PSWQ)

The PSWQ is a 16-item measure of the frequency and intensity of worry that yields a single score [28]. The PSWQ is a single factor structure and has good predictive validity [29].

#### 2.4.5. Reactions to Relaxation and Arousal Questionnaire (RRAQ)

The RRAQ is a nine-item factor analytically derived measure of fear of relaxation [30]. Participants rate the applicableness and accuracy of each item from 1 (not at all) to 5 (very much so). This measure has high retest reliability and strong convergent and discriminant validity [31].

### 2.5. Dependent Outcome Measures

#### 2.5.1. Depression Anxiety Stress Scales-Short Form (DASS-SF)

The DASS-SF [32] is a 21-item self-report questionnaire yielding separate scores for depression, anxiety, and stress over the previous week. This measure has good to excellent psychometric properties [33].

#### 2.5.2. Health Assessment Questionnaire Disability Index (HAQ-DI)

This questionnaire is an assessment for patients with RA where patients report the amount of difficulty they have performing specific activities (dressing and grooming, arising, eating, walking, hygiene, reach, grip, and common daily activities). Each question is scored from 0 to 3 based on whether the patient has no difficulty with the activity (0) or the activity cannot be done at all (3). The construct, convergent, and predictive validity and sensitivity to change have also been established in numerous observational studies and clinical trials [34]. The HAQ-DI was scored with the standard scoring methods whereby the highest subcategory score from each category was used, the use of aids/devices or help was adjusted for, and the summed category scores were divided by the number of categories answered.

#### 2.5.3. Pain Anxiety Symptom Scale (PASS-20)

The PASS-20 is designed to measure fear of pain. This measure includes 4 subscales: avoidance, cognitive anxiety, fearful thinking, and physiological anxiety. PASS-20 has demonstrated good psychometric properties and is highly correlated with its longer version [35].

#### 2.5.4. Pain Catastrophizing Scale (PCS)

The PCS contains 13 items assessing the tendency to misinterpret and exaggerate the threat value of pain sensations. It has good psychometric properties and includes 3 main factors: rumination, magnification, and helplessness [36].

#### 2.5.5. 36-Item Short Form Health Survey (SF-36)

The SF-36 is a 36-item self-report measure that assesses eight domains of health related quality of life. These domains include the following: (1) limitations in physical functioning; (2) social limitations due to emotional or physical troubles; (3) role limitations due to physical health problems; (4) role limitations due to emotional health problems; (5) general mental health; (6) bodily pain; (7) vitality; (8) general health perceptions [37]. The SF-36 has acceptable psychometric properties [38]. The SF-36 can also be scored based on physical and mental components; the current study used individualized scores for each subscale for consistency in using total or subscale scores.

### 2.6. Statistical Analysis

A two-step cluster analysis was performed using SPSS 23 to identify and classify observations into two or more mutually exclusive groups, where members of the groups share properties in common. Five dispositional trait-like variables were used to cluster the observations: experiential avoidance, fear of relaxation, anxiety sensitivity, perfectionism, and worrying based on the AAQ, RRAQ, ASI, FMPS, and PSWQ measures, respectively. The log-likelihood distance measure was used to compute likelihood distance between clusters with subjects assigned to the cluster leading to the largest likelihood. No restrictions were set for the number of clusters and the Bayesian information criterion was used to judge adequacy of the final solution. Differences in sample demographic characteristics were compared according to cluster membership using independent samples *t*-tests and *χ* ^2^ tests for categorical variables in order to characterize differences between the resulting clusters. A multivariate analysis of covariance (MANCOVA) was conducted on outcome measures including mood (DASS-SF), pain catastrophizing (PCS), fear of pain (PASS), quality of life (SF-36), and disability (HAQ-DI) according to cluster membership. Any significant difference on demographic characteristics between clusters was entered as covariates in the MANCOVA. Pairwise comparisons were conducted with Bonferroni adjustment. SPSS version 23.0 (Chicago, IL) was used for all tests performed, with the significance level set at alpha 0.05 and all tests were two-tailed.

## 3. Results

**Figure 1:**
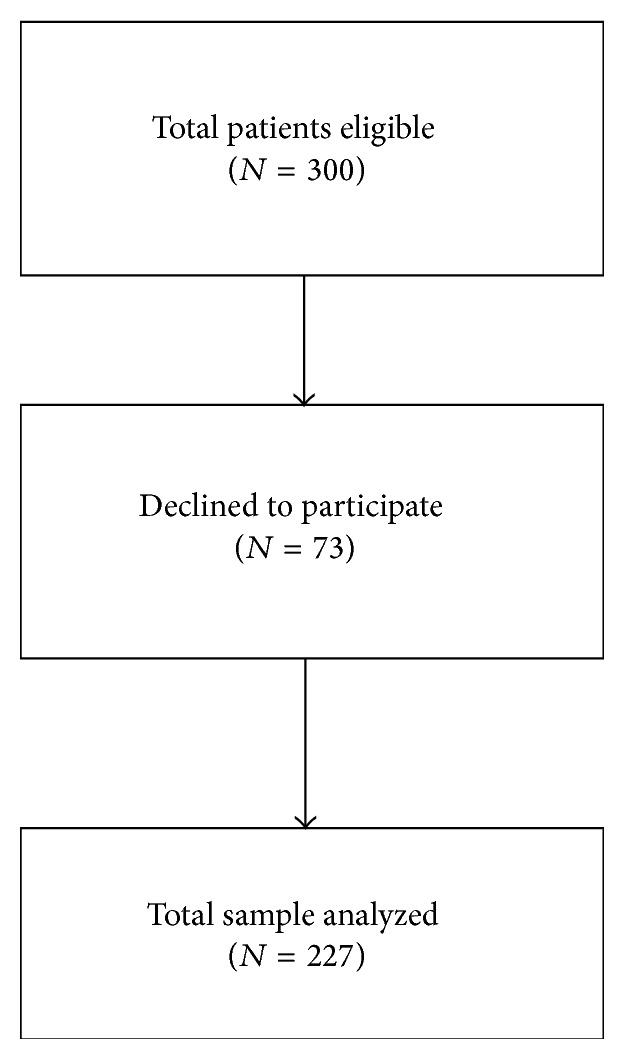
Participant Flowchart.

A total of 300 individuals with RA were eligible for inclusion, of which 227 agreed to participate in the study and completed the questionnaires (Figure 1). The mean age of the sample was 57.8 (SD = 14.4) and the majority of participants were females (75.7%).  Table 1 shows additional sociodemographic and clinical characteristics of the sample.

The two-step cluster analysis of personality questionnaires was conducted with no exclusion of cases. The cluster analysis resulted in an optimal grouping of two clusters (change in Schwartz's Bayesian criterion = −152.1; distance measures ratio = 3.0). The two clusters significantly differed from each other on all clustering variables (see Table 1). Cluster 1 (*n* = 85) was characterized by a dispositional affect comprised of patients scoring significantly higher on experiential avoidance (EA), fear of relaxation (RRAQ), anxiety sensitivity (ASI), perfectionism (FMPS), and worrying (PSWQ) as compared to Cluster 2 (*n* = 142). Demographic characteristics were compared between the two clusters, where it was found that patients in Cluster 1 had been diagnosed with RA for a significantly greater number of years than patients in Cluster 2 (*p* = 0.006). The remaining demographic variables: age, sex, education, relationship status, and average pain intensity were comparable between the two clusters (Table 1).

The two clusters were compared through a MANCOVA while controlling for mean time since RA diagnosis with Bonferroni correction. Pairwise comparisons revealed significant differences between Cluster 1 and Cluster 2 for all mood (DASS-SF), catastrophizing (PCS), and pain anxiety sensitivity (PASS) subscales. Cluster 1 reported significantly higher scores on these measures of distress and cognitive aspects related to pain. There were no significant differences between the clusters for quality of life or disability (see Table 2).

## 4. Discussion

The present study aimed to determine if patients with RA could be differentiated based on dispositional affect. Our second aim was to determine if mood, pain catastrophizing, fear of pain, disability, and quality of life varied as a function of these patient groupings. Participants were divided into two meaningful clusters that represented one group (Cluster 1) composed of patients who reported significantly higher scores on all dispositional variables measured, including experiential avoidance, fear of relaxation, anxiety sensitivity, perfectionism, and worrying, while the second cluster of patients (Cluster 2) included those who scored significantly lower on each of these personality measures. Results also confirmed that mood, pain catastrophizing, and fear of pain measures systematically varied based on patient reports of dispositional variables studied, with those in Cluster 1 demonstrating significantly worse scores on mood, pain catastrophizing, and fear of pain compared to Cluster 2, while controlling for differences in demographic variables between clusters. There were no significant differences found between clusters on disability or quality of life measures.

Our findings revealed that the subset of patients with RA in our sample who reported higher scores on a number of dispositional variables experienced worse mood including increased depressive, anxiety, and stress symptoms, as well as increased cognitions of pain catastrophizing and fear of pain, as shown through higher scores on each pain catastrophizing and pain anxiety symptom subscale. Our results suggest that patients with RA who present with increased endorsement for the cluster of dispositional variables measured within our study may represent a group of patients who experience increased distress, pain catastrophizing, and fear of pain when living with their chronic health condition. Notably, the subset of patients reporting increased endorsement for dispositional affect encompassed fewer patients (*n* = 85) than the cluster of patients who reported levels of these factors (*n* = 142) closer in line to normative means and community samples [39–41]. However, this group of patients endorsing a complex set of dispositional characteristics and increased difficulties in mood, pain catastrophizing, and fear of pain represents a large number of patients with RA experiencing psychological concerns (37% of our sample). This prevalence of patients is comparable to other samples of patients with chronic pain, specifically fibromyalgia, where one study found that 32% of patients displayed elevated mood difficulties, increased pain catastrophizing, and low levels of perceived control over pain [42].

Specific trait-like characteristics including experiential avoidance, fear of relaxation, anxiety sensitivity, perfectionism, and worrying have been linked to a variety of negative outcomes in patients with chronic pain [12, 13, 15, 16, 42]. Patients with RA in Cluster 1 of our sample scored significantly higher on each of these dispositional variables which have been associated to poor mood, catastrophizing, worse functionality, and subjective state of health [43–45].

A number of studies have considered aspects of personality in patients with chronic pain, yet no studies have demonstrated how patients can be clustered together in subgroups based on scoring patterns on a variety of dispositional variables within patients diagnosed with RA experiencing chronic pain. Two previous studies have clustered patients with fibromyalgia based on neurobiological, personality, psychological, and cognitive characteristics. In the first study, cluster analyses classified 97 patients based on anxiety, depression, catastrophizing, control over pain, pain threshold, and multiple random-staircase pressure-pain sensitivity determination [46]. Three subsets of patients were identified through cluster analysis. When considering psychological and cognitive factors from these results, one group was characterized by patients with the highest levels of anxiety, depression, catastrophizing, and the lowest levels of control over pain. Of the remaining two clusters, one scored moderately on all variables while the other had the lowest scores on anxiety, depression, catastrophizing, and the highest control over pain [46]. It was hypothesized that the cluster with the highest levels of anxiety, depression, catastrophizing, and low control over pain may represent the common presentation of fibromyalgia in tertiary care settings. Furthermore, within this study, quality of life (subscales of SF-36) did not significantly differ between clusters. Similarities between our findings and Giesecke et al. (2003) are present whereby Cluster 1 of our sample was comprised of patients who reported significantly greater symptoms of anxiety, depression, and catastrophizing in comparison to Cluster 2. There was also no difference between our clusters of patients on the SF-36 subscales. The SF-36 measures a number of factors; thus, it may not reflect large enough differences in patient distress to differ between subgroups of fibromyalgia [46] or RA patients. Furthermore, the lack of difference in quality of life and similarly in disability between the clusters may be due to the cross-sectional nature of the current study. It may be that time has a strong influence on these two factors and a longitudinal study is needed to capture this effect. Mehta et al. [16] conducted a longitudinal study examining the effect of dispositional traits such as AS and EA on long-term disability among individuals with chronic pain. The study found that those individuals with high levels of dispositional variables had significantly higher levels of long-term disability compared to those with lower levels of dispositional affect [15].

A second study clustered 774 patients with fibromyalgia, some of which were experiencing chronic pain and a comorbid rheumatic disorder [42]. Cluster analysis was used to group patients based on personality traits (neuroticism, extraversion, agreeableness, openness to experience, and conscientiousness). This study divided patients into two clusters. The first cluster was characterized by maladaptiveness whereby patients in this cluster were described as being more likely to experience affective distress and poorly manage social conflicts. These patients scored significantly higher on neuroticism and lower on extraversion, openness to experience, agreeableness, and conscientiousness in comparison to the second cluster [42]. Multivariate analyses comparing the two clusters found that the first cluster, characterized by maladaptiveness, had significantly higher scores for depression, anxiety, and each pain catastrophizing subscale. These significant differences between clusters depression, anxiety, and the pain catastrophizing rumination subscale were also present at six-month follow-up [42]. Our results are generally in line with Torres et al. (2013) findings as our study also resulted in two patient groups where the cluster that endorsed higher levels of dispositional affect also exhibited increased distress, pain catastrophizing, and fear of pain. Specifically, Cluster 1 of our sample and Torres et al. (2013) reported significantly higher scores of depression, anxiety, and all pain catastrophizing subscales suggesting lower mood and the use of ruminative styles that have been associated with magnifying the threat of pain and feeling helpless [12] in both our sample of RA patients and the study of fibromyalgia patients.

Our study contributes to the increased interest of researchers to investigate dispositional affect and trait-like features simultaneously, to present clusters of personality factors rather than considering variables in isolation from one another. Our results provide an understanding of how mood and cognitions associated to pain (pain catastrophizing and fear of pain) may be impacted by a number of dispositional variables within patients with RA. Considering subgroups of patients with RA characterized by dispositional affect had not been previously studied, yet specific personality factors have been associated with psychopathology and difficulties coping in other patient samples [12, 47, 48]. While treatment plans are individualized, intervention studies have found that patients with RA experiencing increased distress benefit from psychological interventions [49, 50]. Providing access to these interventions could allow for targeted approaches to manage poor mood and problematic coping strategies which may be used by patients reporting high scores on the identified dispositional variables. Furthermore, interventions could be developed and targeted to address distinct clusters of patients with RA and within other chronic illnesses. The development of screening tools has been one approach suggested to initiate the assessment and subsequent treatment of psychological comorbidity in patients with RA [12, 50]. Activity pacing is another pain management strategy that may be applied to RA patients who demonstrate specific patterns of dispositional affect. Pacing has been recommended for patients with chronic pain who tend to display obsessive personality traits including psychological inflexibility, fear of relaxation, perfectionism, and experiential avoidance [18]. However, in a small sample of overactive chronic pain patients, applying pacing strategies and enacting behaviour change was difficult when only education of pacing was provided [18].

Specific limitations should be considered when interpreting the findings from this study. First, there are inherent limitations when using a cross-sectional design which inhibit causal relationships to be determined. Second, the personality factors considered were based on a number of different outcome measures rather than one specific personality measure such as the NEO Five-Factor Inventory and thus did not encompass all relevant variables that have been previously studied and linked to mood, with chronic pain. Nonetheless, the dispositional affect measures administered allowed for the analysis of a potentially challenging combination of variables. Further, an important limitation to consider when interpreting our findings is a lack of objective measure of inflammation and thus the inability for inflammation differences between patients to be adjusted for within analyses. Additionally, though the chronicity of pain was controlled for in the MANCOVA, the study demonstrated a significant difference between the two clusters in chronicity of pain. Hence, it may be that the groups differed from each other not only on the dispositional factors but also on this demographic factor. Finally, sample selection bias cannot be ruled out as our sample was recruited from a single site tertiary RA clinic which may compromise the generalizability of our findings.

## 5. Conclusions

In conclusion, the present study identified subgroups of patients with RA based on a number of dispositional variables. The cluster characterized by significantly greater reports of dispositional affect were comprised of RA patients who experienced significantly more depression, anxiety, and stress symptoms in addition to heightened pain anxiety/fear of pain and pain catastrophizing. Ensuring that patients have access to qualified providers of appropriate multimodal treatment may be beneficial for patients with RA experiencing specific difficulties associated with their pain or adjustment including distress, pain catastrophizing, and fear of pain. Clinicians should consider that patients with specific dispositional affect may benefit from referrals for additional social support and programs that target the range of factors included in our study, beginning when they are diagnosed with RA to promote positive adjustment. Future research replicating our findings within RA patients and other samples of chronic pain patients should be carried out so that management programs can be developed to address specific needs of patients such as improving moods and decreasing ruminative styles such as pain catastrophizing and fear of pain.

## Figures and Tables

**Table 1:** Demographic and clinical characteristics of study sample and cluster subgroups.

	Study population	Cluster 1	Cluster 2	*p *(between clusters)
*N*	227	85	142	
Mean age (SD)	57.8 (14.4)	58.3 (14.7)	57.5 (14.4)	0.672
Sex (male %)	24.3	24.7	24.1	0.512
Mean years of education (SD)	13.0 (3.3)	12.5 (3.6)	13.3 (3.1)	0.094
Mean years since RA diagnosis	13.2 (11.0)	15.7 (12.2)	11.6 (9.9)	**0.006**
Relationship status (%)				0.841
Single	11.1	9.5	12.1	
Married or in a serious relationship	74.7	76.2	73.8	
Divorced, separated, widowed	14.2	14.3	14.2	
Average pain intensity	3.8 (2.2)	4.0 (2.0)	3.7 (2.3)	0.092
AAQ (SD)	28.3 (7.5)	32.7 (6.1)	25.7 (7.0)	**0.001**
RRAQ (SD)	12.8 (5.4)	17.0 (5.8)	10.2 (3.1)	**0.001**
ASI total (SD)	15.2 (10.8)	23.8 (10.8)	10.0 (6.9)	**0.001**
FMPS total	74.0 (16.0)	86.1 (13.9)	66.7 (12.5)	**0.001**
PSWQ (SD)	40.9 (12.9)	51.2 (12.4)	34.7 (8.9)	**0.001**

Significant values are shown in bold.

AAQ: Acceptance and Action Questionnaire; ASI: Anxiety Sensitivity Index; FMPS: Frost Multidimensional Perfectionism Scale; HAQ: Health Assessment Questionnaire; PSWQ: Penn State Worry Questionnaire; RRAQ: Reactions to Relaxation and Arousal; SD: standard deviation.

**Table 2:** MANCOVA adjusted for years since RA diagnosis between clusters subgroups.

	Cluster 1 mean (SE)	Cluster 2 mean (SE)	Mean difference between Cluster 1 and Cluster 2 (SE)	*p*
*Disability and quality of life*				
HAQ total	1.08 (0.97)	1.08 (0.97)	0.03 (0.10)	0.749
SF-36 physical functioning	19.63 (0.62)	18.95 (0.49)	0.68 (0.80)	0.540
SF-36 role physical	5.63 (0.18)	5.35 (0.14)	0.29 (0.23)	0.123
SF-36 bodily pain	6.27 (0.23)	6.25 (0.18)	0.02 (0.30)	0.912
SF-36 general health	15.82 (0.30)	15.96 (0.24)	−0.13 (0.39)	0.894
SF-36 vitality	15.08 (0.26)	15.59 (0.20)	−0.52 (0.33)	0.269
SF-36 social function	5.96 (0.31)	6.35 (0.24)	−0.39 (0.40)	0.433
SF-36 role emotional	4.73 (0.15)	4.70 (0.11)	0.03 (0.19)	0.489
SF-36 mental health	20.99 (0.25)	21.33 (0.20)	−0.35 (0.32)	0.331
SF-36 reported health	2.78 (0.10)	2.99 (0.8)	−0.21 (0.12)	0.209

*Distress and coping*				
DASS depression	4.78 (0.34)	2.43 (0.26)	2.4 (0.4)	**0.001**
DASS anxiety	5.88 (0.39)	3.28 (0.30)	2.6 (0.5)	**0.001**
DASS stress	5.22 (0.33)	2.60 (0.25)	2.6 (0.4)	**0.001**
PASS escape avoidance	11.00 (0.62)	8.07 (0.48)	2.9 (0.8)	**0.001**
PASS cognitive anxiety	11.01 (0.60)	6.74 (0.47)	4.3 (0.8)	**0.001**
PASS fearful thinking	7.53 (0.61)	3.19 (0.47)	4.3 (0.8)	**0.001**
PASS physiological anxiety	5.64 (0.46)	2.69 (0.36)	2.9 (0.6)	**0.001**
PCS rumination	11.20 (0.53)	8.11 (0.41)	3.1 (0.7)	**0.001**
PCS magnification	6.24 (0.23)	4.43 (0.17)	1.8 (0.3)	**0.001**
PCS helplessness	11.15 (0.41)	7.92 (0.31)	3.2 (0.5)	**0.001**

Significant values are shown in bold.

DASS: Depression Anxiety Stress Scale; HAQ-DI: Health Assessment Questionnaire-Disability Index; PASS: Pain Anxiety Sensitivity Scale; PCS: Pain Catastrophizing Scale; RA: rheumatoid arthritis; SE: standard error; SF-36: 36-Item Short Form Health Survey.

## Summary

Patients with rheumatoid arthritis often experience chronic pain secondary to their diagnosis. Compared to the general population, patients with rheumatoid arthritis report increased symptoms of depression, anxiety, and stress. Our findings demonstrate that a subgroup of patients with rheumatoid arthritis who score higher on a set of dispositional variables experience significantly greater mood impairment, pain anxiety sensitivity, and pain catastrophizing, representing a combination of psychological aspects that may inhibit patients' adjustment to rheumatoid arthritis. Identifying risk factors that negatively impact patient coping can aid in the design of future interventions.
